# Performance of anthropometric indicators as predictors of metabolic syndrome in Brazilian adolescents

**DOI:** 10.1186/s12887-018-1030-1

**Published:** 2018-02-07

**Authors:** Raphael Gonçalves de Oliveira, Dartagnan Pinto Guedes

**Affiliations:** 1 Universidade Estadual do Norte do Paraná (UENP), Centro de Ciências da Saúde. Alameda Padre Magno, 841, Nova Alcântara, Jacarezinho, PR CEP: 86.400-000 Brazil; 2 Universidade Norte do Paraná (UNOPAR), Centro de Pesquisa em Ciências da Saúde. Rua Marselha, 591, Bairro Piza, CEP: 86.041-140 Londrina, PR Brasil

**Keywords:** Anthropometry, Obesity, Adiposity, Prediction, Diagnosis, Accuracy

## Abstract

**Background:**

It is not clear which is the best anthropometric indicator to predict metabolic syndrome (MetS) in adolescents. Our objective was to identify the predictive power, with respective cut-off points, of anthropometric indicators associated with the quantity and distribution of body fat for the presence of MetS and to determine the strength of the association between the proposed cut-off points and MetS in adolescents.

**Methods:**

The sample consisted of 1035 adolescents (565 girls and 470 boys) aged between 12 and 20 years. Four anthropometric indicators were considered: waist circumference (WC), body mass index (BMI), waist-height ratio (WHtR), and conicity index (C-Index). MetS was defined according to the criteria of the *International Diabetes Federation*. Predictive performance was described through analysis of *Receiver Operating Characteristi*c (ROC) curves with a 95% confidence interval. The most accurate cut-off points were identified through sensitivity, specificity and Area Under the Curve (AUC) values.

**Results:**

The four anthropometric indicators presented significant AUCs close to 0.70. At younger ages (12-15 years) the girls presented a statistically greater capacity to discriminate MetS; however, at more advanced ages (16-20 years) both sexes presented similar AUCs. Among the anthropometric indicators investigated, regardless of sex and age, the WHtR showed the highest discriminant value for MetS, while the C-Index demonstrated a significantly lower capacity to predict MetS. The AUCs equivalent to WC and BMI did not differ statistically. The proposed cut-off points for WHtR (12-15 years = 0.46, 16-20 years = 0.48) presented the highest values of sensitivity and specificity, between 60% and 70%, respectively.

**Conclusion:**

Considering that the best AUC was found for WHtR, we suggest the use of this anthropometric indicator, with the cut-off points presented herein, for the prediction of MetS in adolescents with characteristics similar to the study sample.

## Background

Metabolic syndrome (MetS) refers to a set of risk factors that, when altered, may increase the chances of developing cardiovascular diseases and diabetes mellitus [1–3]. The risk factors include: excess abdominal fat, high blood pressure and triglyceride rates, and altered high density lipoproteins and glycemia [3].

The literature presents strong evidence that cardiometabolic alterations, manifested in adulthood, result from complex interactions between a variety of risk factors that may originate in childhood and adolescence [4, 5]. Therefore, young people who eventually present MetS, with advancing age, tend to be more predisposed to the onset of cardiovascular disease and diabetes mellitus. Thus, early detection of the presence of MetS in the young population is defined as an important primary care strategy that can effectively contribute to the prevention of cardiometabolic outcomes in adulthood and reduce public health expenditures.

However, as the diagnosis of MetS involves invasive laboratory tests to determine the plasma lipid profile and glycemic rate, its inclusion on a large scale in the routine monitoring of the health status of adolescents is complex. In this sense, efforts have been directed in the attempt to indicate more affordable and inexpensive alternatives for epidemiological tracking and, thus, direct specific procedures to those at risk of developing MetS [6–10].

Results found in previous studies suggest that excess body fat is characterized as an important contributor to triggering MetS in the pediatric population [11–13]. In this context, several anthropometric indicators have been proposed to make inferences about body fat profiles. In epidemiological surveys, the body mass index (BMI) is the most widely used anthropometric indicator and offers indications related to the total quantity of body fat [14]. In addition, waist circumference (WC), waist-height ratio (WHtR), and the conicity index (C-Index) have been proposed and successfully tested to measure cardiometabolic risk in young people due to their ability to provide estimates of centripetal concentration of body fat [15, 16].

Considering that the anthropometric indicators associated with the quantity and distribution of body fat are easy to handle, are not invasive and have low cost, it is important to identify the predictive power of each anthropometric indicator and its respective cut-off points for detection of MetS in young population. However, it should be pointed out that this referral does not seek to replace medical intervention, since it does not exclude the need to identify the individual components to confirm the diagnosis of MetS. Screening, when performed in environments with high concentrations of young people, such as schools, can reach a high number of adolescents, especially those who have difficulty accessing or do not attend the health system. Thus, once the adolescents most likely to present MetS have been identified, they can be referred for specialized medical follow-up.

Some studies have evaluated the predictive capacity of anthropometric indicators to detect MetS; however, conflicting data were found. Kelishadi et al. [6] found WC to be the best predictor, followed by BMI and WHtR. In contrast, in the study by Jung et al. [7] BMI was the best predictor, followed by WC and WHtR. Nambiar et al. [8] found WHtR to be a better predictor compared to BMI, while WC did not demonstrate significant predictive capacity. Benmohammed et al. [9] found better predictive capacity for WHtR, followed by WC and BMI.

In addition to the divergences found among the results of the studies, it is noteworthy that the C-Index has not been tested for its predictive ability to detect MetS in adolescence. In adults the C-Index presented superior performance to other anthropometric indicators to predict cardiovascular risk [17].

Therefore, the objectives of the present study were to identify the predictive power, with respective cut-off points, of four anthropometric indicators associated with the quantity and distribution of body fat (WC, BMI, WHtR, and C-Index) for the presence of MetS and to determine the strength of the association between the proposed cut-off points and MetS in adolescents.

## Methods

The study is linked to a larger project, which aimed to identify the prevalence of MetS and associated factors in adolescents. For this, a descriptive cross-sectional survey was carried out with a population base involving schoolchildren from the city of Jacarezinho, Paraná, Brazil. Data collection extended from August to November 2014. The intervention protocols used followed the Declaration of Helsinki and were approved by the Research Ethics Committee of the Universidade Norte do Paraná – UNOPAR (Opinion 1.302.963).

### Sample and selection of subjects

The reference population included adolescents of both sexes, between 12 and 20 years of age, enrolled in public and private elementary schools (6th to 9th grade) and high school (1st to 3rd grade). Initially, the sample size was established to meet the primary objective of the project to identify the prevalence of MetS and associated factors, assuming a 95% confidence interval, a sampling error of 3 percentage points, and an increase of 10% to allow for eventual lost cases during data collection. In addition, considering that the sample planning involved conglomerates, we defined the effect of the sample design (*deff*) as equivalent to 1.5, estimating, therefore, a minimum sample of 1000 adolescents in school. However, the final sample used in the treatment of information was composed of 1035 adolescents (565 girls and 470 boys)**.** In the present study, the statistical power of the sample, stratified by sex (565 girls and 470 boys), was calculated a posteriori and enabled identification with 80% power, a significance of 5%, and areas under the ROC (Receiver Operating Characteristic) curve of at least 0.53 and 0.56 for girls and boys, respectively.

Regarding the selection of the subjects, we aimed to obtain probabilistic sampling by clusters, having as a reference gender, year of study, and period in which the adolescents were enrolled in each stratum of the school structure (public and private). The criteria adopted to exclude some adolescents drawn for the study were: (a) refusal to participate in the study; (b) not signing the Free and Informed Consent Form; (c) any health problem that temporarily or permanently prevented participation in the study; (d) using any type of medication that could induce changes in the study variables; (e) undergoing any type of specific diet; (f) pregnancy; and (g) non-attendance at school on the day scheduled to begin data collection. In these cases, a new draw was carried out to restore any sample losses.

### Anthropometric indicators

In order to determine the body weight measurements, an anthropometric scale with a 10 g definition was used, brand SECA (Berlin, Germany), model 813, checked every ten weighings, while to carry out the height measurements an aluminum stadiometer was used with a 1 mm scale, brand SECA (Berlin, Germany), model 213. The WC measurements were performed using a flexible anthropometric inelastic fiberglass tape with a 1 mm scale, brand SECA (Berlin, Germany), model 203.

Measurements of body weight, height, and WC were performed according to the recommendations of the World Health Organization [18]. Each previously trained researcher performed the same function during the data collection period in order to minimize possible measurement errors. To measure body weight, the adolescent, barefoot and wearing minimal clothing, was positioned standing in the center of the scale platform, upright, with arms beside the body and looking at a fixed point in front of them.

For the height measurements, the adolescent, barefoot, was placed on the base of the stadiometer, upright, with the upper limbs hanging beside the body, feet together, trying to maintain the posterior surfaces of the heels, pelvic girdle, shoulder girdle, and occipital region in contact with the measurement scale. The distance between the plantar region and the vertex was determined with the aid of a cursor. The adolescent remained in inspiratory apnea and their head was oriented in the Frankfurt plane parallel to the ground.

WC measurements were determined with the adolescent standing, with a relaxed abdomen and arms beside the body. The anthropometric tape was positioned in the horizontal plane, so as to encircle the natural waist line, at the coincident point of the mean distance between the last costal arch and the iliac crest, in a firm manner; however, without skin compression. The reading was obtained at the end of a normal expiration.

The BMI was calculated through the ratio between the body weight measured in kilograms and the height expressed in meters squared (kg/m^2^). The WHtR was obtained by dividing the waist circumference measure by the height in centimeters [19]. The C-Index was defined by the equation [20]:

$$ Conicity\ index\ \left(C\hbox{--} Index\right)=\frac{waist\ circumference\ (m)}{0.109\ \sqrt[\kern1em ]{\frac{body\ weight\ (Kg)}{height\ (m)}}} $$

### Metabolic syndrome

MetS was identified by analyzing the blood content of plasmatic lipids (triglycerides and high density lipoproteins - HDL-cholesterol) and blood glucose, resting blood pressure (systolic and diastolic), and abdominal fat accumulation (waist circumference), according to the criteria proposed by the International Diabetes Federation (IDF) [4]. In this case, MetS is defined by the presence of a high waist circumference (< 16 years: both sexes ≥ Percentile 90, ≥ 16 years: boys ≥ 90 cm and girls ≥ 80 cm) and at least two other compromised components: increased triglycerides (≥ 150 mg/dL), decreased HDL-cholesterol (< 16 years: both sexes < 40 mg/dL, ≥ 16 years: boys < 40 mg/dL and girls < 50 mg/dL), high fasting blood glucose (≥ 100 mg/dL), and altered blood pressure (systolic ≥ 130 mmHg or diastolic ≥ 85 mmHg).

Plasmatic lipid and blood glucose measurements were performed by collecting 10 ml venous blood samples at the elbow crease after a 10-12 h fasting period between 07:00 and 08:00 a.m. The serum was immediately separated by centrifugation, and the HDL-cholesterol dosages were determined by the precipitating reactive method, serum triglycerides by the enzymatic glycerol method, and glycemia by the calorimetric enzymatic methodology.

The systolic and diastolic arterial blood pressure levels were measured by the auscultatory method using a mercury column sphygmomanometer. With the adolescent sitting, after a minimum of 5 min of rest, blood pressure was measured in the left arm. The systolic blood pressure value corresponded to Korotkoff phase I and diastolic blood pressure to phase V, or the disappearance of sounds. Two measures were taken, considering the mean value of both measures for calculation purposes.

### Statistical treatment

Statistical analysis was performed using SPSS software, version 22. For the analysis of continuous variables, procedures of descriptive statistics were used (mean ± standard deviation). As the treated variables presented normal distribution of data, the comparisons between sex (girls and boys) and age (12 to 15 years and 16 to 20 years) for the anthropometric indicators were performed using *two-way* analysis of variance with interaction, accompanied by the Scheffe multiple comparison test.

The predictive capacity, sensitivity, and specificity of the four anthropometric indicators (WC, BMI, WHtR, and C-Index) to identify the presence of MetS, accompanied by the respective 95% confidence intervals, were defined using the ROC curve, to establish cut-off points in diagnostic or screening tests [21]. The Area Under the ROC Curve (AUC) was used specifically to determine the predictive capacity of the anthropometric indicators. In this case, an AUC = 1 indicates perfect predictive power, while AUC ≤ 0.5 indicates that predictive power is not better than chance. For purposes of interpretation, the confidence interval equivalent to AUC allows determination of whether the predictive ability of the anthropometric indicator is significant, and therefore, its lower limit should not be less than 0.50. The complexity of the sample was considered in order to estimate the parameters.

Cut-off points for each anthropometric indicator capable of predicting MetS were determined by the best balance between sensitivity and specificity. Thus, the main objective of the analysis is to determine the value at which sensitivity and specificity indicate a threshold that maximizes the true-positive rate, maintaining the lowest possible rate of false-positive cases. Possible significant differences between the properties of sensitivity, specificity, and AUCs were identified using McNemar’s statistical test [22].

After determination of the cut-off points for each of the predictive anthropometric indicators of MetS, they were dichotomized based on their respective reference values. The prevalence ratios accompanied by the respective 95% confidence intervals, stratified by sex and age, were calculated using Poisson regression.

## Results

### Sample characteristics

Statistical information equivalent to the anthropometric indicators that characterize the sample selected for the study are provided in Table 1. The boys were statistically heavier and taller than the girls. When comparing the mean values for the anthropometric indicators that reflect the body fat distribution pattern (WC, WHtR, and C-Index), the older boys and adolescents presented significantly higher scores. Regarding BMI, the scores found showed a significant increase with advancing age; although similar in both sexes. The presence of MetS was identified in 4.5% of the sample, being significantly higher in boys (5.2% versus 3.9%) and older adolescents (4.9% versus 4.2%). According to the diagnostic criteria based on BMI proposed by *International Obesity Task Force* [23], the excess body weight (overweight and obesity) was identified in 21,3% of the sample, showing no significant difference between girls and boys (22.2% and 20.3%, respectively), but significantly higher in older adolescents (19.4% versus 23.2%).

**Table 1:** Mean, standard deviation, and F statistic values for anthropometric measurements and indicators associated with excess weight/body fat

		Age		F test		
		12–15 Years	16–20 Years	Sex	Age	Interaction
Height (cm)	Girls	158.19 ± 8.52	162.21 ± 5.26	40.110	31.224	19.625
	Boys	163.50 ± 9.01	173.69 ± 6.89	*p* < 0.001	*p* < 0.001	*p* < 0.001
Body weight (kg)	Girls	53.49 ± 12.37	59.44 ± 14.56	37.437	28.575	20.717
	Boys	57.80 ± 11.91	69.27 ± 14.40	*p* < 0.001	*p* < 0.001	*p* < 0.001
Waist circumference (cm)	Girls	67.82 ± 8.94	71.85 ± 9.19	23.608	9.910	0.672
	Boys	72.09 ± 9.14	77.80 ± 9.98	*p* < 0.001	*p* < 0.001	ns
Body mass index (kg/m^2^)	Girls	20.27 ± 4.27	22.32 ± 4.23	1.645	5.635	0.579
	Boys	20.63 ± 4.07	23.38 ± 4.07	ns	*p* = 0.004	ns
Waist/Height ratio	Girls	0.42 ± 0.05	0.44 ± 0.08	4.912	5.862	2.374
	Boys	0.43 ± 0.03	0.46 ± 0.06	*p* = 0.022	*p* = 0.001	ns
Conicity index	Girls	1.07 ± 0.04	1.10 ± 0.08	8.092	6.735	3.214
	Boys	1.11 ± 0.06	1.13 ± 0.06	*p* < 0.001	*p* < 0.001	*p* = 0.041

*ns* not significant

### Anthropometric and MetS indicators

**Fig. 1:**
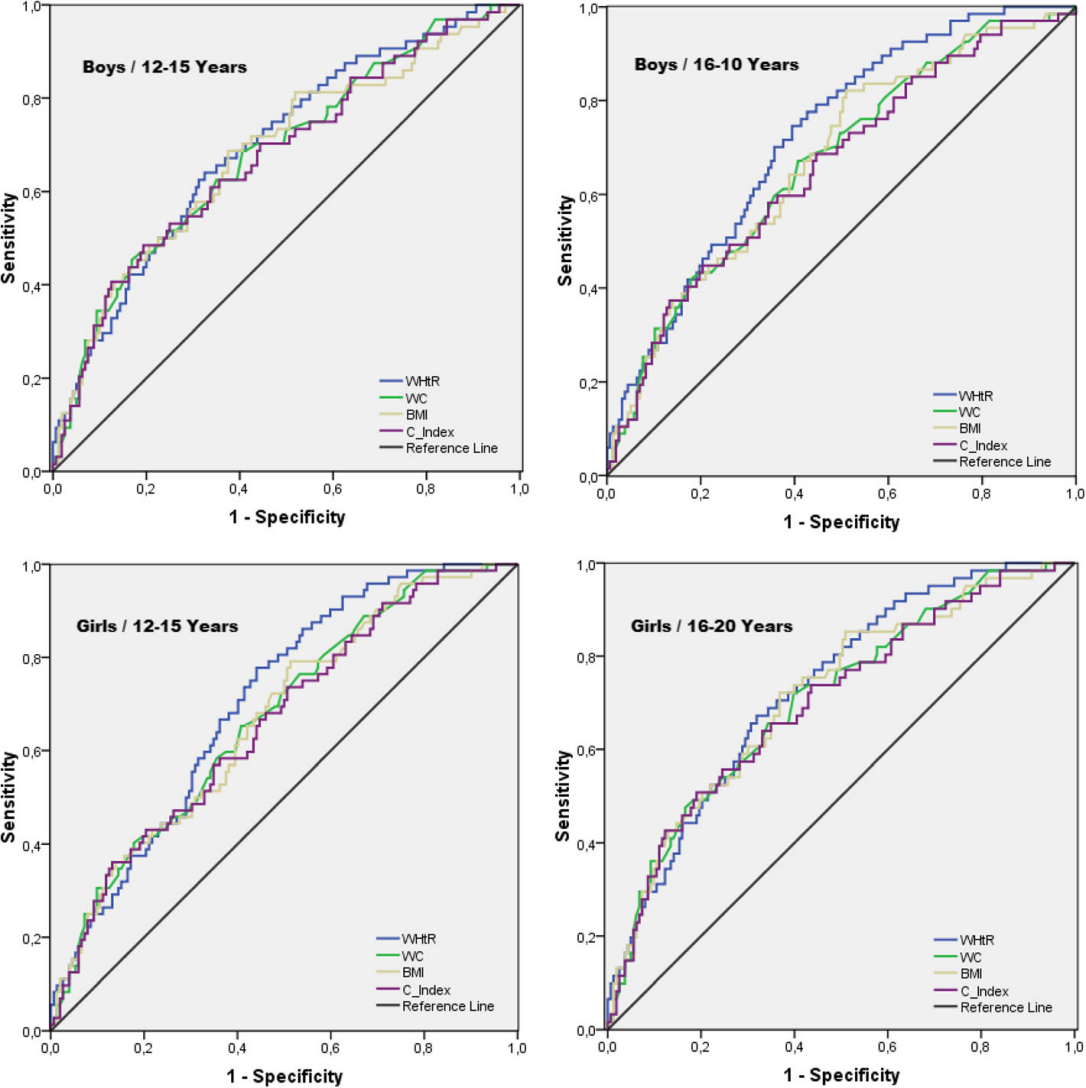
ROC plots for the predictions of metabolic syndrome by anthropometric indicators: waist-height ratio (WHtR), waist circumference (WC), body mass index (BMI), and conicity index (C-Index)

The performance of the anthropometric indicators as predictors of MetS is presented in Table 2 and Fig. 1. The values of sensitivity and specificity with the most appropriate balance between them are presented for the four anthropometric indicators as discriminators of MetS. It was noted that, regardless of sex and age, WHtR demonstrated better sensitivity and specificity to discriminate MetS. However, the four anthropometric indicators presented significant AUCs, close to 0.70. At younger ages (12-15 years) girls presented a statistically larger ability to discriminate MetS; however, at more advanced ages (16-20 years) both sexes presented similar AUCs. Among the anthropometric indicators investigated, the C-Index presented significantly lower MetS prediction capacity, whereas WHtR presented the highest discriminant value for MetS. The AUCs equivalent to WC and BMI did not differ statistically.

**Table 2:** Performance of anthropometric indicators as predictors of metabolic syndrome

	Sensitivity (CI_95%_)		Specificity (CI_95%_)		Area under the curve (CI_95%_)	
	12–15 years	16–20 years	12–15 years	16–20 years	12–15 years	16–20 years
Waist circumference						
Girls	61.2 (55.1–67.5)	66.7 (59.9–73.9)	62.1 (56.7–67.7)	67.2 (61.1–73.6)	0.70 (0.66–0.75)	0.73 (0.68–0.78)
Boys	57.4 (51.9–63.2)	62.5 (56.4–68.3)	57.9 (53.0–63.1)	63.2 (56.9–69.5)	0.66 (0.61–0.72)	0.71 (0.66–0.77)
χ^2^ Test	1.984 (ns)	2.663 (ns)	2.486 (ns)	2.495 (ns)	4.183 (*p* = 0.032)	2.137 (ns)
Body mass index						
Girls	62.9 (57.0–68.9)	67.8 (61.5–74.3)	62.3 (57.2–67.5)	67.4 (61.4–73.6)	0.71 (0.67–0.76)	0.73 (0.67–0.79)
Boys	58.8 (53.1–64.7)	63.1 (57.2–69.2)	59.3 (53.8–64.9)	64.2 (59.1–69.4)	0.67 (0.62–0.73)	0.72 (0.67–0.78)
χ^2^ Test	2.417 (ns)	5.011 (*p* = 0.043)	1.438 (ns)	1.612 (ns)	4.258 (*p* = 0.028)	1.738 (ns)
Waist/Height ratio						
Girls	65.9 (59.7–72.3)	69.7 (63.4–76.2)	66.2 (60.5–72.0)	70.1 (64.2–76.2)	0.73 (0.68–0.79)	0.76 (0.71–0.82)
Boys	60.7 (54.8–66.8)	64.5 (58.6–70.6)	61.1 (55.9–66.5)	65.3 (60.4–70.4)	0.69 (0.65–0.74)	0.74 (0.70–0.79)
χ^2^ Test	5.846 (*p* = 0.015)	6.101 (p < 0.001)	5.312 (*p* = 0.031)	5.152 (*p* = 0.044)	4.496 (*p* = 0.018)	2.471 (ns)
Conicity index						
Girls	60.2 (54.4–66.2)	62.6 (56.7–68.6)	60.4 (55.7–65.3)	64.1 (59.9–68.5)	0.68 (0.64–0.72)	0.71 (0.67–0.76)
Boys	55.6 (49.8–61.5)	58.4 (52.9–64.1)	55.8 (50.3–61.2)	60.0 (59.1–64.2)	0.64 (0.60–0.69)	0.68 (0.63–0.73)
χ^2^ Test	4.973 (*p* = 0.047)	2.542 (ns)	4.814 (*p* = 0.049)	2.531 (ns)	3.879 (*p* = 0.042)	2.989 (ns)

*ns* not significant, *CI 95%* confidence interval of 95%

After determination of the cut-off points, the strength of the association between each of the anthropometric indicators and the presence of MetS was verified. The prevalence ratios and their respective confidence intervals are presented in Table 3. The four anthropometric indicators investigated presented significant and positive associations with MetS. The boys who presented WC and BMI scores higher than the cut-off points defined in the present study were approximately one-and-a-half to two times more likely to present MetS, while girls, under these same conditions, were around two to three times more likely to present MetS. Specifically in the case of WHtR, boys and girls with scores higher than the cut-off points found herein presented probabilities two and three times greater, respectively, of presenting MetS.

**Table 3:** Cut-off points with higher accuracy and prevalence ratios between anthropometric indicators and metabolic syndrome in adolescents

	Cut-off points		Prevalence ratio (CI_95%_)	
	12–15 years	16–20 years	12–15 years	16–20 years
Waist circumference				
Girls	75.8	78.1	2.38 (1.87–3.11)	2.61 (1.88–3.58)
Boys	77.2	83.3	1.41 (1.13–1.80)	1.56 (1.20–1.99)
Body mass index				
Girls	21.4	23.1	2.89 (2.07–4.21)	3.02 (2.03–4.46)
Boys	21.5	23.9	1.52 (1.25–1.89)	1.79 (1.43–2.23)
Waist/Height ratio				
Girls	0.46	0.48	3.13 (2.09–4.30)	3.51 (2.43–4.79)
Boys	0.46	0.48	1.79 (1.27–2.50)	2.06 (1.42–2.95)
Conicity index				
Girls	1.13	1.16	1.72 (1.18–2.53)	2.18 (1.42–3.11)
Boys	1.16	1.20	1.29 (1.07–1.61)	1.42 (1.17–1.78)

*CI 95%* confidence interval of 95%

## Discussion

The present study investigated the ability of anthropometric indicators associated with the quantity and distribution of body fat to discriminate the presence of MetS in adolescents. The ability of the four anthropometric indicators to predict MetS in adolescents aged 12 to 20 years of both sexes was confirmed. However, when comparing the AUCs found for each of the anthropometric indicators, significant differences were identified, indicating different accuracy. The anthropometric indicator that showed the highest predictive capacity for MetS was WHtR, followed, in this order, by BMI, WC, and C-Index.

It is not uncommon to find higher scores equivalent to anthropometric indicators associated with quantity (BMI) and centripetal body fat distribution (WC, WHtR, and C-Index) in boys and older adolescents [6, 9, 10, 24]. With advancing age, adolescents become more susceptible to the endocrine effects triggered by pubertal development, which impact differently and significantly on the greater accumulation and pattern of body fat distribution [25, 26].

Corroborating with findings made available through a systematic review that synthesized data from approximately 100 surveys conducted in different regions of the world [27], the present study identified a higher prevalence of MetS in boys and older adolescents. Using the same diagnostic criteria (IDF), the MetS prevalence observed was higher than that found recently in the Brazilian young population (4.5% vs 2.6%); however, close to that found in cities in the same geographic region (4,1%) [28]. When compared with international data, the proportion observed in the present study is lower than that reported in North American and European adolescents; however, higher than that found in adolescents from Asian countries [29]. On this note, it is emphasized that the IDF diagnostic criterion is intended to minimize false-positive cases and therefore, presents more conservative cut-off points, as well as considering WC as a mandatory component to identify MetS. Therefore, when compared to other diagnostic criteria adapted for use in adolescents, the IDF criterion should indicate a lower prevalence of MetS [30].

In the present study, with identical cut-off points for both sexes, although different for adolescents aged 12-15 years (0.46) and 16-20 years (0.48), the WHtR was indicated as the anthropometric indicator that best discriminates the presence of MetS. Both cut-off points indicated sensitivity and specificity values between 60% and 70%, which moderately minimizes false-positive and false-negative cases. However, a very uncommon way of analyzing the diagnostic capacity of specific cut-off points is by calculating the positive (PLR) and negative (NLR) likelihood ratios. In the case of the youngest group (12-15 years), the PLR was equivalent to 1.95 in girls and 1.86 in boys, suggesting that those adolescents with WHtR ≥0.46 may present approximately twice the chance of a positive diagnosis being true; while the NLR corresponded to 0.52 and 0.48, respectively, which is also close to twice the chance of a negative diagnosis confirming the absence of MetS. Among the older adolescents (16-20 years), The PLR was equivalent to 2.33 and 1.86, while the NLR corresponded to 0.43 and 0.54 for girls and boys, respectively.

The study by Benmohammed et al. [9] using the IDF criteria for diagnosis of MetS in Algerian adolescents also found a better predictive capacity through the use of WHtR. However, it is noteworthy that, regardless of the anthropometric indicator used (WHtR, WC, or BMI), a high accuracy was identified (AUC ≥ 90), with all cut-off points indicating maximum sensitivity (100%) and specificity, around 75%.

On the other hand, coinciding with the findings of the present study, other investigations have found predictive capacity for MetS through anthropometric indicators associated with the quantity and distribution of body fat. Jung et al. [7], also using the IDF criteria to diagnose MetS in German adolescents, observed moderate to high accuracy (0.83 ≥ AUC ≤ 0.88) for the anthropometric indicators investigated, with an advantage for BMI followed by WC and WHtR. However, as a limitation, the sample involved few adolescents and only boys. Kelishadi et al. [6] using a less rigorous diagnostic criteria to diagnose MetS in adolescents (de Ferranti criteria), also found a moderate to high predictive capacity (0.72 ≥ AUC ≤ 0.89) for the anthropometric indicators considered; however, to the advantage of WC, followed by BMI and WHtR.

The disagreements between the studies that have been carried out regarding the anthropometric indicator with better predictive performance for MetS may perhaps be justified by the ethnic origin of the adolescents selected in the different studies. In this regard, the ethnicity of adolescents is capable of influencing the definition of the cut-off points of the anthropometric indicators associated with overweight and body fat and the individual components that make up MetS, which could impact on their prevalence [4]. Thus, identification of the most appropriate anthropometric indicator and its respective cut-off points capable of predicting a higher risk for MetS may be dependent on the geographical location in which the study is performed. In addition, it should be taken into account that MetS can affect adolescents for reasons other than just excess fat and body weight. MetS may be caused by behavioral issues such as inadequate eating habits, excessive sedentary time, and physical inactivity [31].

Referring specifically to the cut-off points best able to predict MetS through the WHtR, a systematic review aimed at analyzing the potential of anthropometric indicators to predict cardiovascular disease indicated WHtR ≥0.50 as the most appropriate for both sexes [32]. This cut-off point is higher than those found in the present study; however, the systematic review composed, mostly, data involving adults, which may explain the higher cut-off point; especially when compared to younger adolescents. In the Italian pediatric population, high sensitivity and specificity for WHtR ≥0.50 were demonstrated in the detection of at least two metabolic or cardiovascular risk factors; however, it was not the purpose of the study to propose and test another cut-off point value [33].

Using the cut-off points defined in the present study the four anthropometric indicators investigated were significantly associated with the presence of MetS. However, we highlight the higher probability of identifying MetS in girls than in boys. Girls with WHtR ≥ 0.46 (12-15 years) and WHtR ≥ 0.48 (16-20 years) demonstrated probabilities around three to three and a half times higher for MetS, while boys with WHtR scores above cut-off points were approximately twice as likely to present MetS. Previous studies have also found greater accuracy to identify MetS by means of anthropometric indicators among girls [6, 9]; however, it is still unclear whether this finding should be attributed to the specific metabolic profile of each sex, or the behavioral implications that differentiate girls and boys at this age.

### Strengths and limitations

To our knowledge this is the first study performed with Brazilian adolescents to verify the performance of different anthropometric indicators associated with the quantity and distribution of body fat as predictors of MetS. Identifying MetS in pediatric populations is not routine in the clinical setting, except in specific situations, such as the presence of obesity and diabetes. In addition, the decrease in the frequency of medical consultations during adolescence reduce the possibility of early detection of metabolic alterations, and the lack of diagnosis, control, and treatment of these alterations may constitute a factor impeding the prevention of future cardiometabolic outcomes. The findings of the present study will enable, by means of simple and accessible procedures, the screening of the possible presence of MetS and, when appropriate, referral to a specialized service to confirm the diagnosis.

On the other hand, the present study presents some limitations that must be considered. School-based sampling may weaken the representativeness of the adolescent population. However, it should be emphasized that adolescents from all the city’s educational networks were distributed proportionally in the schools selected for the study. Even taking into account the low refusal rate for anthropometric measurements and laboratory tests (≈ 8%), the possibility of selection bias cannot be ruled out, since it was not possible to compare the treated indicators between the adolescents participating and not participating in the study. It is also important to note that, since there is no universal criterion for defining MetS, we chose to use the criterion proposed by the IDF and, in this sense, estimates of the prevalence of MetS may vary according to the criterion used.

## Conclusions

The four anthropometric indicators investigated demonstrated ability to predict MetS in adolescents aged 12 to 20 years of both sexes. However, considering that the best AUC was found for the WHtR, we suggest the use of this anthropometric indicator, with the cut-off points presented herein, for the prediction of MetS in adolescents with similar characteristics to the study sample. In this sense, it is assumed that approximately three out of four adolescents with MetS can be correctly diagnosed, constituting, therefore, a reasonable alternative to be used in initial screening to identify adolescents at higher cardiometabolic risk.

## Abbreviations

AUC: Area Under the Curve
BMI: Body mass index
C-Index: Conicity index
IDF: International Diabetes Federation
MetS: Metabolic syndrome
NLR: Negative likelihood ratio
PLR: Positive likelihood ratio
ROC: Receiver Operating Characteristic
WC: Waist circumference
WHtR: Waist-height ratio
